# Application of plasma polymerized pyrrole nanoparticles to prevent or reduce de-differentiation of adult rat ventricular cardiomyocytes

**DOI:** 10.1007/s10856-021-06595-7

**Published:** 2021-09-09

**Authors:** Omar Uribe-Juárez, Rafael Godínez, Juan Morales-Corona, Myrian Velasco, Roberto Olayo-Valles, M. C. Acosta-García, E. J. Alvarado, Luis Miguel-Alavez, Oscar-J. Carrillo-González, María G. Flores-Sánchez, Roberto Olayo

**Affiliations:** 1 Departamento de Ingeniería Eléctrica, Universidad Autónoma Metropolitana, Av. San Rafael Atlixco 186, Col. Leyes de Reforma 1ra Secc., Del. Iztapalapa, C. P. 09340, Ciudad de México, México; 2 Departamento de Física, Universidad Autónoma Metropolitana, Av. San Rafael Atlixco 186, Col. Leyes de Reforma 1ra Secc., Del. Iztapalapa, C. P. 09340, Ciudad de México, México; 3 Departamento de Neurodesarrollo y Fisiología, División de Neurociencias, Instituto de Fisiología Celular, Universidad Nacional Autónoma de México, Av. Universidad 3000, Col Ciudad Universitaria, Del. Coyoacán, C. P. 04510, Ciudad de México, México; 4 Departamento de Biología de la Reproducción, Universidad Autónoma Metropolitana, Av. San Rafael Atlixco 186, Col. Leyes de Reforma 1ra Secc., Del. Iztapalapa, C. P. 09340, Ciudad de México, México; 5 Facultad de Ingeniería, Vicerrectoría de Investigación, Universidad La Salle México, Benjamín Franklin 45, Col. Condesa, Del. Cuauhtémoc, C. P. 06140, Ciudad de México, México

## Abstract

Cardiovascular diseases are the leading cause of death in the world, cell therapies have been shown to recover cardiac function in animal models. Biomaterials used as scaffolds can solve some of the problems that cell therapies currently have, plasma polymerized pyrrole (PPPy) is a biomaterial that has been shown to promote cell adhesion and survival. The present research aimed to study PPPy nanoparticles (PPPyN) interaction with adult rat ventricular cardiomyocytes (ARVC), to explore whether PPPyN could be employed as a nanoscaffold and develop cardiac microtissues. PPPyN with a mean diameter of 330 nm were obtained, the infrared spectrum showed that some pyrrole rings are fragmented and that some fragments of the ring can be dehydrogenated during plasma synthesis, it also showed the presence of amino groups in the structure of PPPyN. PPPyN had a significant impact on the ARVC´s shape, delaying dedifferentiation, necrosis, and apoptosis processes, moreover, the cardiomyocytes formed cell aggregates up to 1.12 mm^2^ with some aligned cardiomyocytes and generated fibers on its surface similar to cardiac extracellular matrix. PPPyN served as a scaffold for adult ARVC. Our results indicate that PPPyN-scaffold is a biomaterial that could have potential application in cardiac cell therapy (CCT).

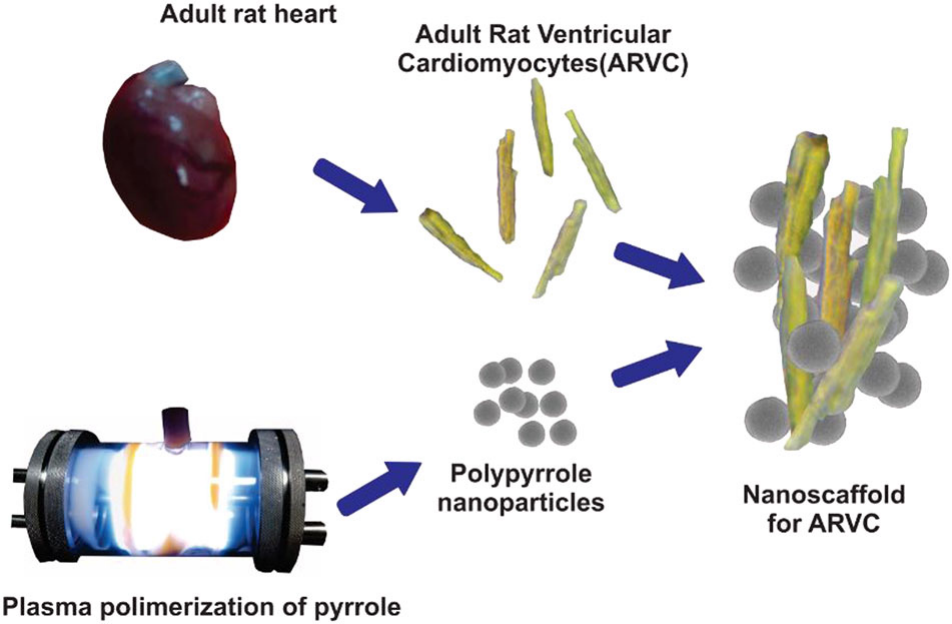

## Introduction

Cardiovascular diseases (CVD) are the leading cause of death in the world, the World Health Organization estimates that 80% of CVD deaths correspond to myocardial infarction and strokes [1, 2]. Although in certain cases heart diseases can be treated, there are currently no treatments to recover damaged heart tissue after a myocardial infarction. Some emerging therapies to repair or delay cell remodeling after a myocardial infarction are pharmacologic treatments [3, 4], and cardiac cell therapy (CCT) [5, 6].

CCT with human pluripotent stem cells, which are until now the only demonstrated and robust source of either authentic human cardiomyogenic progenitors or differentiated cardiomyocytes, has been shown to enhances cardiac function in macaque monkeys with large myocardial infarctions, and their clinical safety in humans are being to be studied [7–9]. Some problems faced in CCT are cell retention, survival of the engrafted cells, cell differentiation, and integration of transplanted cells with the tissue [9, 10]. Some of these problems can be overcome using scaffolds for cardiac cells [11]. Scaffolds are biomaterials that provide a healthy environment for cell adhesion, differentiation, proliferation, and migration [12].

Plasma polymerized pyrrole (PPPy) is a biomaterial that has been used to coat surfaces improving adhesion of different cell types [13, 14]. Cell adhesion is essential for the formation, structure, and integration of a tissue [15]. Cell adhesion to PPPy-coated surfaces is associated in part with the amino groups present in the PPPy chemical structure [16, 17], since amino groups promote cell adhesion [18, 19].

The interaction of cardiomyocytes with the extracellular matrix (ECM) is mainly mediated by integrins [15], and computational simulations have shown that PPPy could interact with integrins [20]. Therefore, it is likely that cardiomyocytes will adhere to PPPy-coated surfaces. The interaction with integrins can influence a wide range of cellular functions including adhesion, spreading, migration, ECM organization, apoptosis, and cell differentiation [21, 22].

Furthermore, cardiomyocytes also interact with neighboring cardiomyocytes and with other cell types, carrying out mechanical and electrical functions in the heart [23]. In tissue engineering of cardiomyocytes, it is, therefore, vitally important that the biomaterial used as a scaffold does not limit or interfere with cellular interactions, and with mechanical or electrical functions. Whether we reduce the scaffold´s size, the cardiomyocytes could interact with each other easier. Due to their size and the PPPy´s cell adhesion, PPPy nanoparticles (PPPyN) could function as a kind of nano scaffold for cardiomyocytes, furthermore, nanoparticles could function as a cellular adhesive, promoting the union between cardiomyocytes, and therefore, the three-dimensional cell aggregates formation or construction.

Nanomaterials are increasingly being used as part of scaffolds in tissue engineering, and regenerative medicine [24, 25]. Although, there are reports of polymeric microparticles (50–100 µm) used as scaffolds for cell therapies [26], up to the date this article was written, we do not know other studies that use nanoparticles as scaffolds. In this work, we show that PPPyN, with sizes in the range of 100–500 nm, interact with adult rat ventricular cardiomyocytes (ARVC) and can be employed as nanoscaffolds for the development of cardiac microtissues.

## Materials and methods

### Reagents

Pyrrole was purchased from Sigma-Aldrich. M199 medium, fetal bovine serum, goat serum, antibiotic with antimycotic and collagenase type II were purchased from GIBCO. Hanks balanced salt solution (HBSS) was prepared with CaCl_2_ (1.26 mM), MgCl_2_ (0.49 mM), MgSO_4_ (0.41 mM), KCl (5.33 mM), KH_2_PO_4_ (0.44 mM), NaHCO_3_ (4.17 mM), NaCl (137.93 mM), Na_2_HPO_4_ (0.33 mM), D-Glucose (5.55 mM) and HEPES (25 mM). HBSS without Ca was prepared with MgCl_2_ (0.42 mM), KCl (5.33 mM), KH_2_PO_4_ (0.44 mM), NaHCO_3_ (4.17 mM), NaCl (137.93 mM), Na_2_HPO_4_ (0.33 mM), D-Glucose (5.55 mM), HEPES (25 mM) and EDTA (0.25 mM). All salts, HEPES, and EDTA were purchased from Sigma-Aldrich.

### Animals

ARVC were isolated from four months old male Wistar rats (280–350 g). Rats were provided by the local animal facility (Universidad Autónoma Metropolitana—Iztapalapa). The rats were kept on 12/12 h light/dark inverted cycle with free access to food and water and were handled in accordance with the Official Mexican Standard NOM-062-ZOO-1999.

### Isolation of ARVC

A rat was anesthetized with an intraperitoneal injection of sodium pentobarbital using a dose of 60 mg/kg. The thorax and abdomen of the rat were shaved and bathed in 70% alcohol. The thorax was opened with the help of scissors. When the heart was exposed, the rat was euthanized by cervical dislocation. The heart was then removed and put in a 25 ml centrifuge tube with 10 ml of cold HBSS without Ca. A cannula was introduced in the aorta and secured with suture. The heart was the perfused using a Langendorff system with 40 ml of HBSS without Ca at 37 °C for 5 min. A second perfusion was made with 40 ml of HBSS with 0.45 mg/ml of collagenase type II at 37 °C for 25 min. The heart was put in a 25 ml centrifuge tube with 10 ml of HBSS with 0.45 mg/ml of collagenase type II on a water bath at 37 °C for 20 min. The heart was then put in a 50 ml glass beaker with 10 ml of cold HBSS where the aorta and atria were removed with surgical scissors. The ventricles were cut into ~1 mm^2^ pieces with help of two scalpels and the ventricles pieces were put in a 25 ml centrifuge tube with 10 ml of cold HBSS and gently shaken by hand for 1 min. The solution of HBSS and ventricles pieces was filtered with a 250 μm nylon mesh. The filtrate was further filtered in a 40 μm cell strainer. The cardiomyocytes captured in the cell strainer were washed with 30 ml of cold HBSS and collected in a petri dish by turning the cell strainer upside down and bathing it with 20 ml M199 medium. The cardiomyocytes were collected in a 25 ml centrifuge tube with 20 ml of M199 medium and centrifuged at 1200 rpm for 5 min. The supernatant was discarded and 5 ml of M199 medium supplemented with 5% goat serum, 5% fetal bovine serum, and 1% antibiotic with antimycotic were added. The cell pellet gently resuspended with a pipette and stored for later use [27].

### Culture

M199 medium supplemented with 5% goat serum, 5% fetal bovine serum, and 1% antibiotic with antimycotic was used as culture medium. ARVC were counted using a Neubauer chamber. Approximately 1 × 10^4^ cardiomyocytes were placed in a 15 ml centrifuge tube with 1 ml of fresh culture medium. Then, 200 μg of PPPyN were added. The tube was centrifuged at 1200 rpm for 5 min. The formed cell button was gently dispersed with a pipette. The last two steps were repeated twice more, and the resulting suspension was placed in a 35 × 10 mm culture dish with 1 ml of fresh culture medium. The same protocol was followed for the control group, but without PPPyN. The medium was changed every 3rd day by removing 1 ml of culture medium from the culture dish. The medium taken from the culture dish was placed in a 5 ml centrifuge tube and centrifuged at 1200 rpm for 5 min. The supernatant was carefully removed, and 1 ml of fresh culture medium was added to the centrifuge tube. The cells were resuspended by gentle stirring with a pipette, and the content of the tube was put back into the culture dish.

### PPPyN synthesis

PPPyN were synthesized in a borosilicate glass cylindrical plasma reactor 12 cm in length, 9 cm outer diameter, and 5 mm thickness. The ends of the reactor were capped by two stainless steel covers through which round stainless-steel electrodes of 7 cm in diameter were inserted. The electrodes were connected to a radio frequency generator (13.56 MHz). One of the covers had two additional openings, one of which was connected to a pressure sensor and the other to a vacuum trap in series with a vacuum pump. The monomer was introduced into the reactor through an opening located in the center of the borosilicate tube. The frequency generator´s power was 45 W, and the pressure inside the reactor was 1.6 Torr.

The infrared spectrum of PPPyN was acquired on an ThermoScientific Nicolet iS5 infrared spectrophotometer equipped with an attenuated total reflectance (ATR) attachment.

### Scanning electron microscopy (SEM)

PPPyN were coated with gold by sputtering and observed in a Jeol 7600F HRSEM scanning electron microscope.

ARVC were prepared for SEM imaging by first placing a sample in a 15 ml centrifuge tube, centrifuging at 1200 rpm for 5 min, and carefully removing the supernatant. Phosphate buffered saline (PBS, 2 ml) with 5% of paraformaldehyde was added to the centrifuge tube. ARVC were incubated 48 h in the paraformaldehyde. ARVC were then washed three times with PBS. The PBS was removed, and 1 ml of osmium tetroxide was added. ARVC were incubated for 2 h in the osmium tetroxide. Afterward, the osmium tetroxide was removed and the ARVC were dehydrated with by washing with solutions of gradually higher ethanol concentration, starting with 20% and going up to 100%. ARVC were then dried in a critical point drier, coated with gold, and observed in a Jeol JSM 5900 LV scanning electron microscope.

### Statistical analysis

The length and width of each cardiomyocyte were measured using ImageJ software. The length:width ratio (*R*_L:W_) of each cardiomyocyte was determined using software Wolfram Mathematica 11. To determine whether the mean *R*_L:W_ of both groups (control and PPPyN) was significantly different, a Student’s *t*-test was performed for independent samples and different variances (*p* < 0.05).

Due to control group data did not have a normal-type distribution, the data of both groups were normalized using the Box–Cox transformation with λ = −1 [28, 29]. Data normality of both groups was verified by the Kolmogorov Smirnov normality test (*p* < 0.1), and again, a Student’s *t*-test was performed for independent samples and different variances (*p* < 0.05), statistical analysis was performed using Wolfram Mathematica 11.

## Results and discussion

**Fig. 1:**
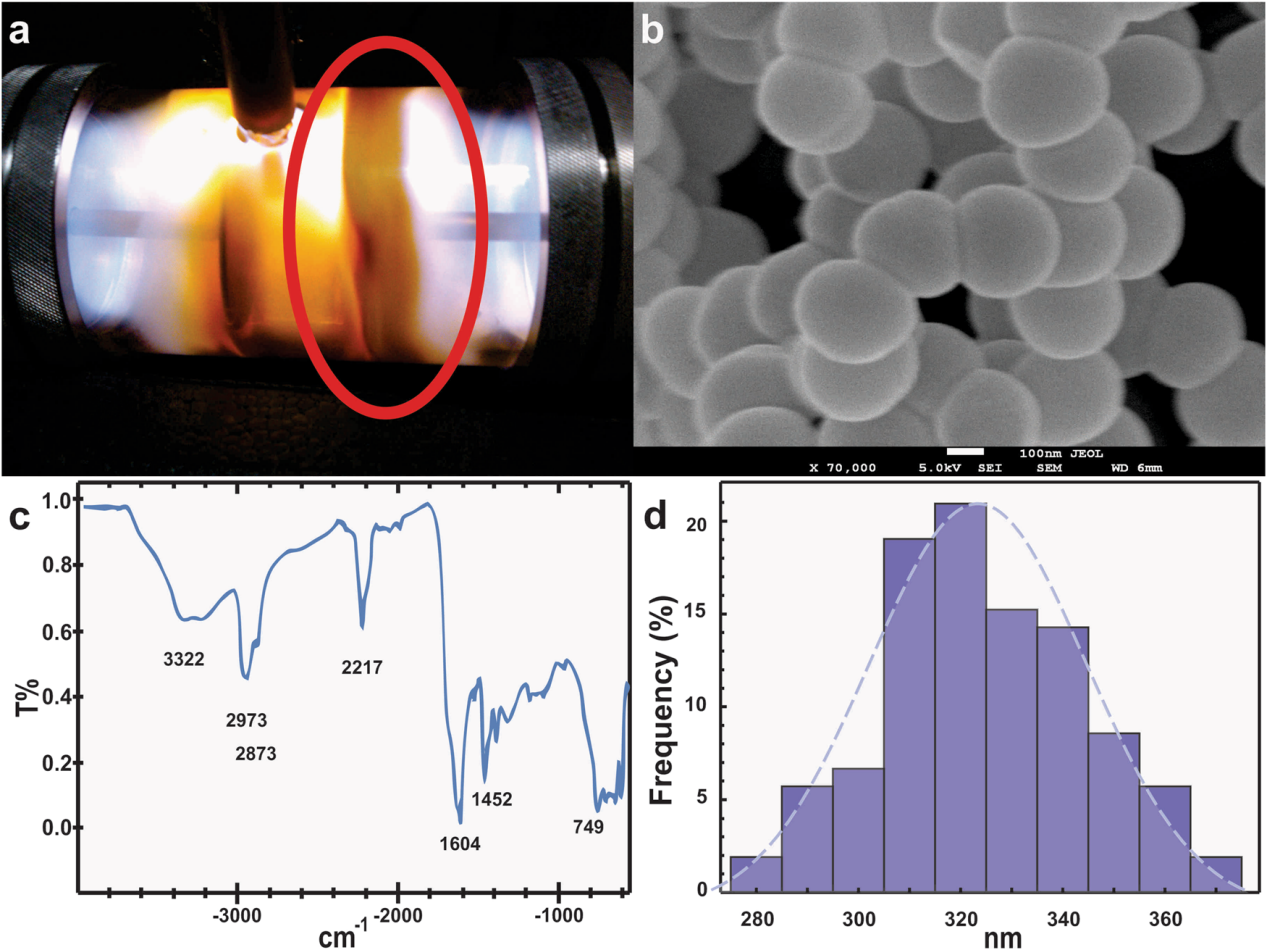
**a** PPPyN (red circle) synthesis in the plasma reactor, **b** SEM image of PPPyN, **c** infrared spectrum of PPPyN, and **d** PPPyN size distribution

PPPyN were synthesized (Fig. 1a) and observed in the scanning electron microscope (Fig. 1b). The PPPyN diameters show a normal-type (Fig. 1d) distribution with a mean diameter of 330 nm and standard deviation of 20 nm (*n* = 105). The PPPyN size decrees linearly with the applied power [30], therefore, smaller PPPyN could be obtained increasing the synthesis power, however, previous studies have shown that nanoparticles with sizes below 100 nm are not suitable for use as nanoscaffolds. For example, polypyrrole nanoparticles with diameters of 100 nm synthesized by oxidative polymerization were endocytosed by IMR90 and J774A1 cells [31]. While some authors suggest that nanoparticle size is not the main factor for endocytosis, it is understood that smaller particles gain entry into the cell more readily [32]. The size of PPPyN obtained in this study is, thus, adequate for the desired application.

In the PPPyN infrared spectrum (Fig. 1c), both the broad band between 3700 and 3000 cm^−1^ centered at 3222 cm^−1^ and the band at 1604 cm^−1^ corresponds to amine groups, the presence of these groups is expected given that the monomer is pyrrole. The broad band centered at 3222 cm^−1^ is also associated with OH groups. The presence of hydroxyl groups is likely due to PPPyN´s oxidation by atmospheric oxygen when the reactor is opened at the end of the synthesis. The band centered at 2937 cm^−1^ corresponds to aliphatic C-H bonds, while the signal at 2217 cm^−1^ indicates the presence of C≡C or C≡N bonds. The presence of these bonds is evidence that the pyrrole rings can be fragmented during the synthesis and the fragments can be dehydrogenated. The signal at 1452 cm^−1^ suggests vibrations in the plane of C=C and C–H bonds. Finally, the band at 749 cm^−1^ could be attributed to the vibration of the skeleton and is indicative of the formation of polymer chains [16, 17, 20, 33].

**Fig. 2:**
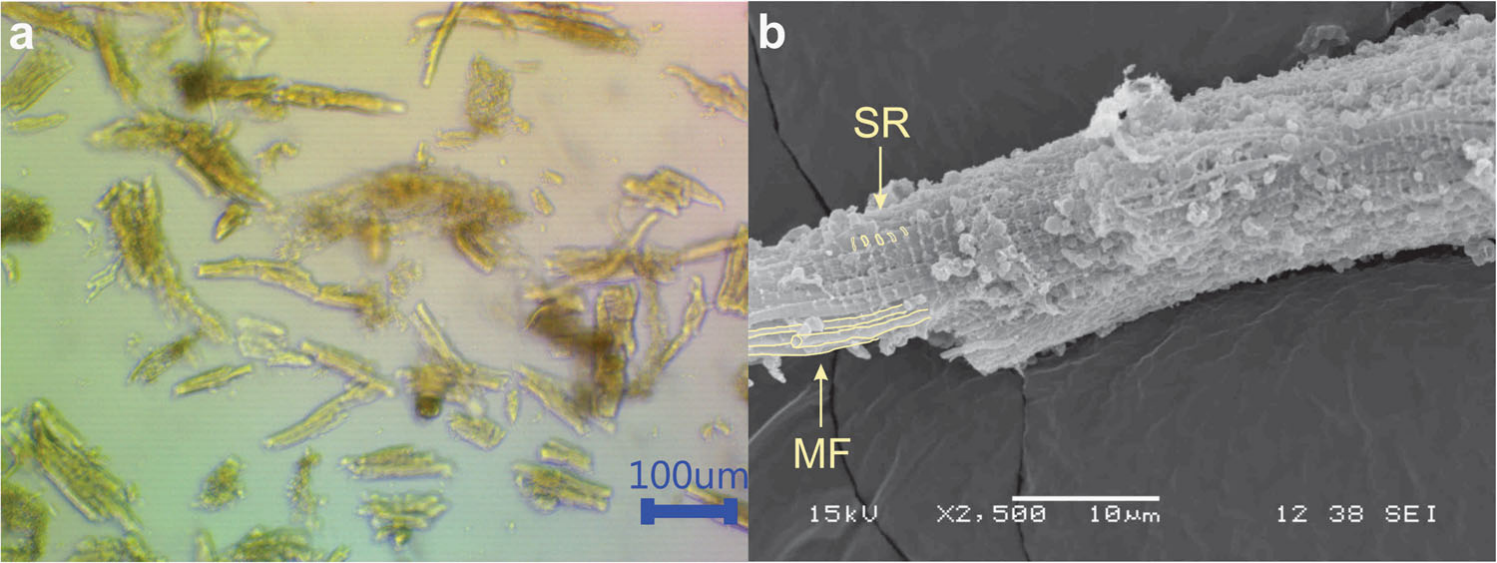
ARVC 1 day of culture **a** optical microscopy **b** SEM image

ARVC were isolated from a murine animal model. The cells had a characteristic rod shape and an approximate length of 100 µm (Fig. 2a) [27, 34]. In the SEM image of ARVC, it is possible to appreciate myofibers (MF) and sarcoplasmic reticulum (SR) (Fig. 2b) [35]. SR forms a continuous network linking the transverse tubular system [36]. The transverse tubular system forms periodic regular arrays that correlate to the position of the Z-lines [36, 37]. The Z-lines are located at the lateral borders of the sarcomere (the fundamental unit of striated muscle) [38]. The sarcomeres of the ARVC could, therefore, be located between two contiguous SR of a myofiber.

**Fig. 3:**
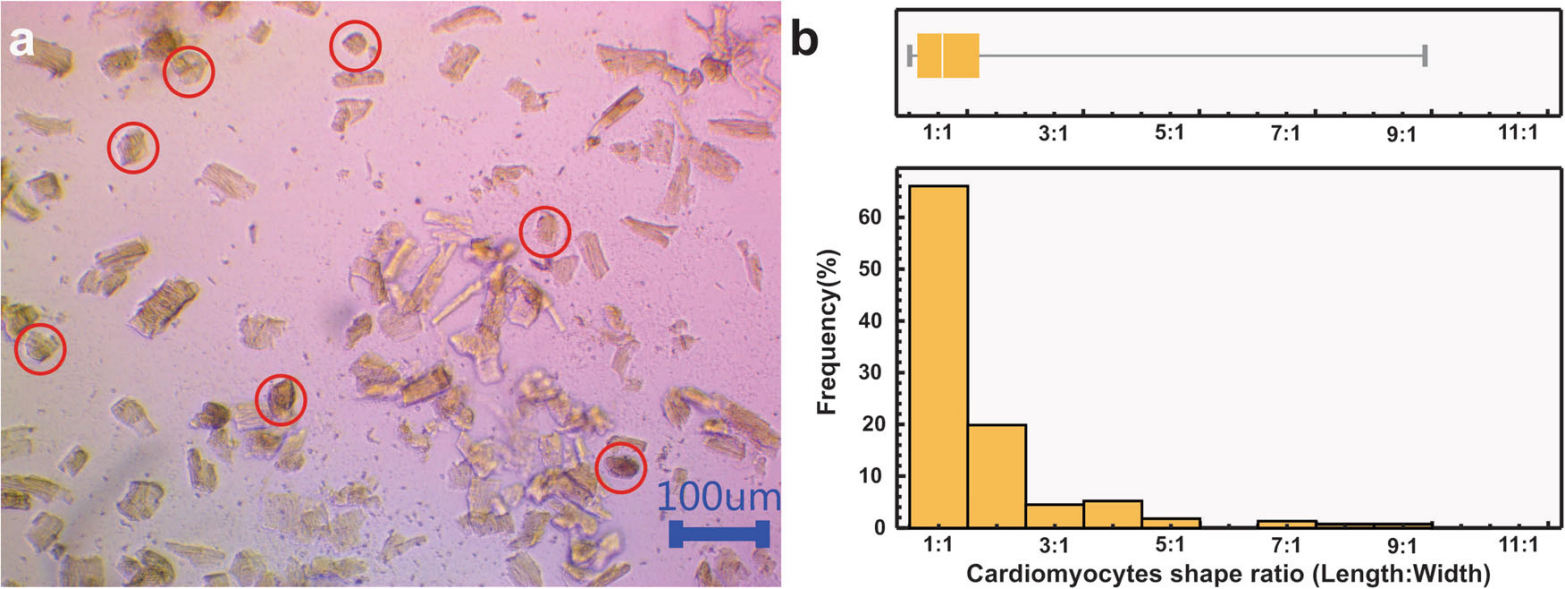
**a** ARVC at 7th day of culture. **b** ARVC shape ratio (*R*_L:W_) histogram and boxplot

At 7th day of culture, the ARVC had been lost their characteristic rod shape and acquired a square or round morphology (Fig. 3a). Cardiomyocyte shape is related to their gene expression. The length/width ratio (*R*_L/W_) has been correlated with cardiomyocyte gene expression [27, 39]. ARVC isolated from healthy rats have *R*_L/W_~7.5 [40]. Changes in the ARVC shape begun to be perceptible at the 3rd day of culture. After 7 days of culture, most ARVC lose their characteristic rod shape and acquire a square or round shape (*R*_L:W_ ~1) [27, 41]. Genes involved in dedifferentiation, necrosis, and apoptosis are upregulated in square cardiomyocytes (*R*_L:W_ = 1) [27, 39]. ARVC life span in culture is limited, although ARVC cultures of up to 15 days have been reported [42, 43], some researchers report that 6–8 days is an optimal time frame for ARVC culture [44, 45]. At 7 days of culture, the mean *R*_L:W_ of the ARVC used in this study was 2, with a standard deviation of 1.4 (*n* = 156). Approximately 86% of the ARVC had a *R*_L:W_ between 1 and 3 (Fig. 3b).

**Fig. 4:**
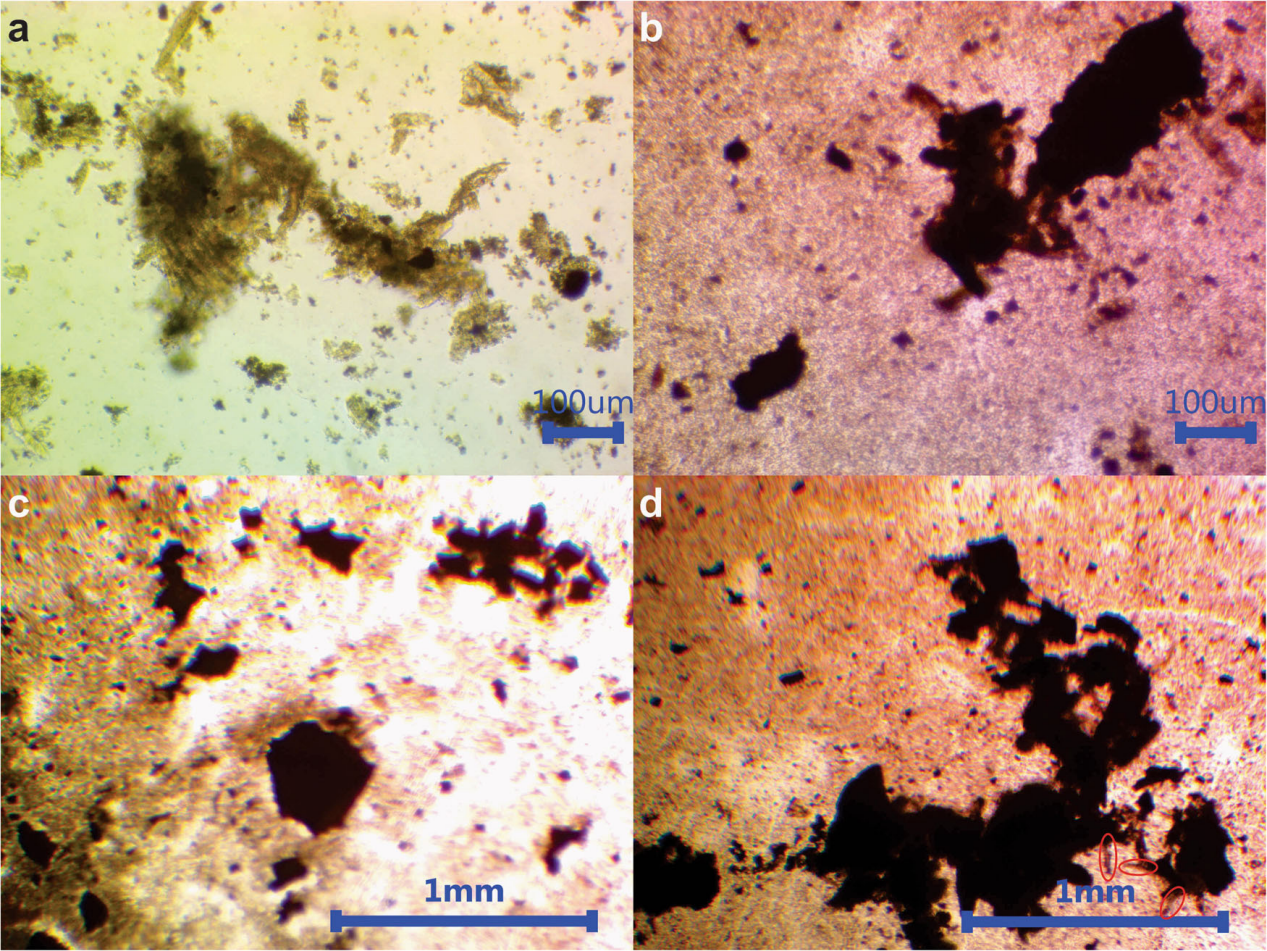
Cardiomyocytes with PPPyN **a** 1 day of culture. **b** 8 days of culture. **c** 15 days of culture. **d** 30 days of culture

ARVC with PPPyN were maintained in culture for up to 30 days. From the 1st day of culture, cardiomyocytes began to adhere to PPPyN (Fig. 4a). This result was expected since cell adhesion to PPPy has been previously reported [13, 14]. Over the next 7 days, ARVC were covered by PPPyN (Fig. 4b). Between the 10th and 12th day, the small cellular aggregates began to bind to each other to form larger cell aggregates (Fig. 4c). Between the 21st and 25th days, the cell aggregates reached their maximum size, and their size remained constant until the 30th day. The largest cell aggregate reached an area of 1.12 mm^2^, some ARVC could be observed in the cellular aggregate periphery (red circles, Fig. 4d). Since it is difficult to see the cell aggregates with optical microscopy, these are best described by SEM.

**Fig. 5:**
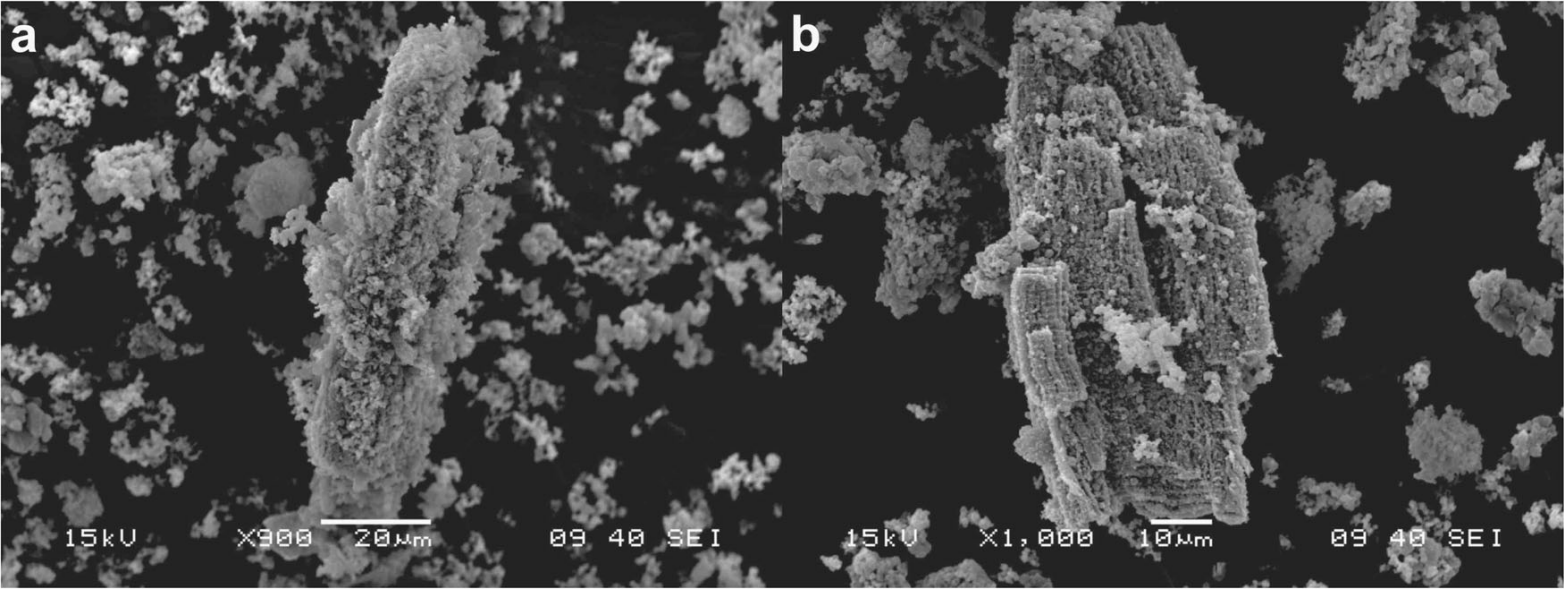
SEM images of ARVC cultured with PPPyN after 30 days of culture. **a** ARVC with PPPyN on its surface. **b** Group of aligned ARVC

SEM images of ARVC culture with PPPyN after 30 days, showed that some ARVC still maintained their characteristic rod shape, and had PPPyN adhered on their surface (Fig. 5a). It is also possible to observe a small group of aligned cardiomyocytes (Fig. 5b). ARVC organization in fibers is of great importance to obtain functional cardiac tissues, and it is still one of the main challenges for the regeneration of cardiac tissue [46, 47].

**Fig. 6:**
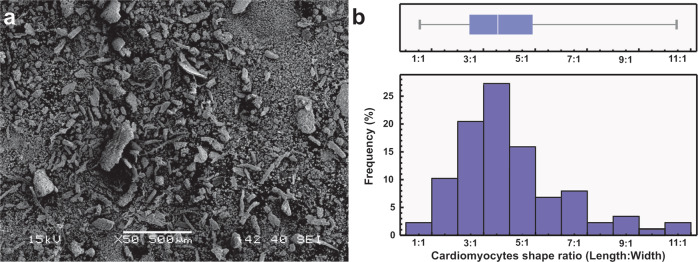
ARVC cultured with PPPyN after 30 days of culture. **a** SEM image of fragmented cellular aggregates, **b** ARVC shape ratio (*R*_L:W_) histogram and boxplot

Although the cellular aggregates were gently fixed and dehydrated for SEM observation, during the critical point drying process cellular aggregates were fragmented. This allowed us to analyze the morphology of the ARVC that formed the cellular aggregates (Fig. 6a). The mean *R*_L:W_ of ARVC cultured with PPPyN was 5 with a standard deviation of 2.12 (*n* = 88), and only 13% of the ARVC had *R*_L:W_ between 1 and 3 (Fig. 6b).

**Fig. 7:**
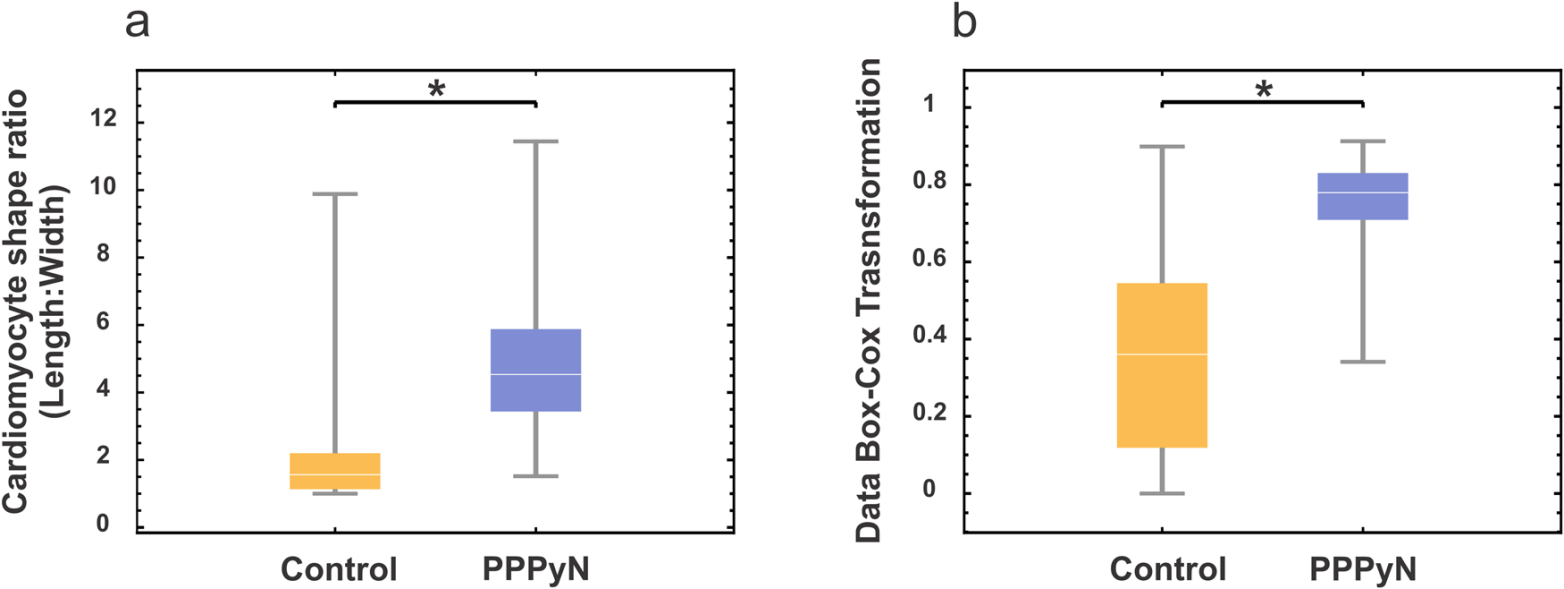
**a** ARVC shape ratio (*R*_L:W_) boxplots, control and PPPyN groups. **b** Boxplots after Box–Cox transformation

The *R*_L:W_ of ARVC cultured with PPPyN after 30 days was significantly different from the control group (*p* < 0.05, Fig. 7a). Data normality of both groups was verified by the Kolmogorov Smirnov normality test. This test determined that control group data did not have a normal-type distribution. For this reason, the data of both groups were normalized using the Box–Cox transformation with λ = −1 [28, 29]. After the Box Cox transformation, the Kolmogorov Smirnov test was performed again, resulting in both groups having a normal-type distribution (*p* < 0.1). Once normality was verified in both groups, a Student’s *t*-test was performed again for independent samples and different variances. The test determined that the means of both groups were significantly different (*p* < 0.05, Fig. 7b). Our results show that, at least up to 30 days, PPPyN had a significant impact on the shape of ARVC and, probably, on their gene expression delaying dedifferentiation, necrosis, and apoptosis processes [27, 39].

**Fig. 8:**
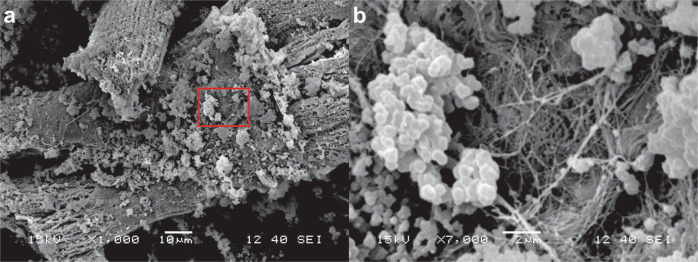
**a** ARVC with fibers on its surface. **b** ARVC surface close-up

Fibers similar to those present in cardiac ECM [48, 49] were observed on the surface of ARVC cultured with PPPyN (Fig. 8a, b). ECM is part of the cardiac tissue and, therefore, to regenerate cardiac tissue it is also necessary to restore the ECM [11, 50]. Since PPPyN could be interacting with integrins [20] and integrins influence a wide range of cellular functions including ECM organization [21, 22], PPPyN could be helping some ARVC begin to express their own ECM.

## Conclusions

ARVC begin to dedifferentiate at the 3rd day of culture [27, 41] and generally do not remain in culture for more than 15 days [42]. PPPyN provided a healthy environment where ARVC could remain attached and differentiated for at least up to 30 days. Therefore, PPPyN served as a scaffold for ARCV. In addition, ARVC cultured with PPPyN formed cell aggregates of up to 1.12 mm^2^. Within the cell aggregates, some cell alignment was observed and some ARVC began to generate what appears to be their own ECM.

Some current challenges in CCT are cell retention in the infarcted myocardium [51], and the development of effective protocols to differentiate cells into mature cardiomyocytes [52]. Cell adhesion to PPPyN could improve cell retention (or cardiac micro-tissue retention) in the infarcted myocardium. Furthermore, PPPyN could help keep the transplanted cells differentiated. For these reasons, PPPyN have potential application in CCT.

In addition to CCT, we are interested in exploring the development of other types of microtissues employing PPPyN nanoscaffolds. In particular, we are interested in the development of skin microtissues employing keratinocytes and PPPyN.
